# Prediction of CO_2_ Permeance across ZIF-L@PDMS/PES Composite Membrane

**DOI:** 10.3390/membranes13020134

**Published:** 2023-01-19

**Authors:** Meor Muhammad Hafiz Shah Buddin, Abdul Latif Ahmad, Muhd Izzudin Fikry Zainuddin

**Affiliations:** 1 School of Chemical Engineering, Universiti Sains Malaysia Engineering Campus, Nibong Tebal 14300, Malaysia; 2 School of Chemical Engineering, College of Engineering, Universiti Teknologi MARA, Shah Alam 40450, Malaysia

**Keywords:** polymeric membrane, gas separation, composite membrane, metal-organic frameworks

## Abstract

The current work predicted the permeance of CO_2_ across a ZIF-L@PDMS/PES composite membrane using two different models. The membrane was fabricated by dipping a PES hollow fiber membrane in a coating solution made using PDMS that contained ZIF-L. First, flat sheet ZIF-L@PDMS membranes were fabricated to verify the role of ZIF-L on the gas separation performance of the membrane. Based on the data, the presence of ZIF-L in the PDMS matrix allowed enhancement of both permeability and selectivity of CO_2_, where the maximum value was obtained at 1 wt% of ZIF-L. The performance of ZIF-L@PDMS layer, as a function of ZIF-L loading, was well-predicted by the Cussler model. Such information was then used to model the CO_2_ permeance across ZIF-L@PDMS/PES composite membrane via the correction factor, which was introduced in the resistance in series model. This work discovered that the model must consider the penetration depth and the inorganic loading (in the case of ZIF-L@PDMS/PES). The error between the predicted CO_2_ permeance and the experimental results was found to be minimal.

## 1. Introduction

Polymeric membranes are a simple yet effective method for gas separation. As the research progress and its formulations evolve, the polymeric membrane can be grouped according to its configuration: symmetric or asymmetric [1]. Specifically, in this study, an asymmetric membrane consisting of two different materials was examined. Polyethersulfone (PES) was coated with poly-dimethoxy silane (PDMS), forming a PDMS/PES composite membrane. To further enhance its performance, ZIF-L (a 2D metal-organic framework) was included in the PDMS layer. The proposed configuration was formulated as an alternative to allow good dispersion of filler in a hollow fiber membrane, rather than a mixed matrix. Dispersion of inorganic fillers in an asymmetric polymeric membrane is often viewed as wasteful because such fillers tend to be located even at the highly porous substructure of the membrane. Hence, locating the filler on the surface was a reasonable option to avoid such issues. This work compared several models to predict the performance of both PDMS/PES and ZIF-L@PDMS/PES composite membranes. Several types of membranes were fabricated in order to gather reliable data for accurate predictions. They were: (i) pristine hollow fiber PES membrane, as the substrate; (ii) flat sheet ZIF-L@PDMS membrane; and (iii) PDMS/PES and ZIF-L@PDMS/PES composites in the form of hollow fiber. Typical models used to estimate the performance of composite membranes require an extension, as the permeance of the ZIF-L@PDMS layer is, at that point, a function of ZIF-L loading. To the author’s knowledge, reports on gas permeance prediction across a composite membrane including inorganic filler in the coating layer are very limited.

**Figure 1:**
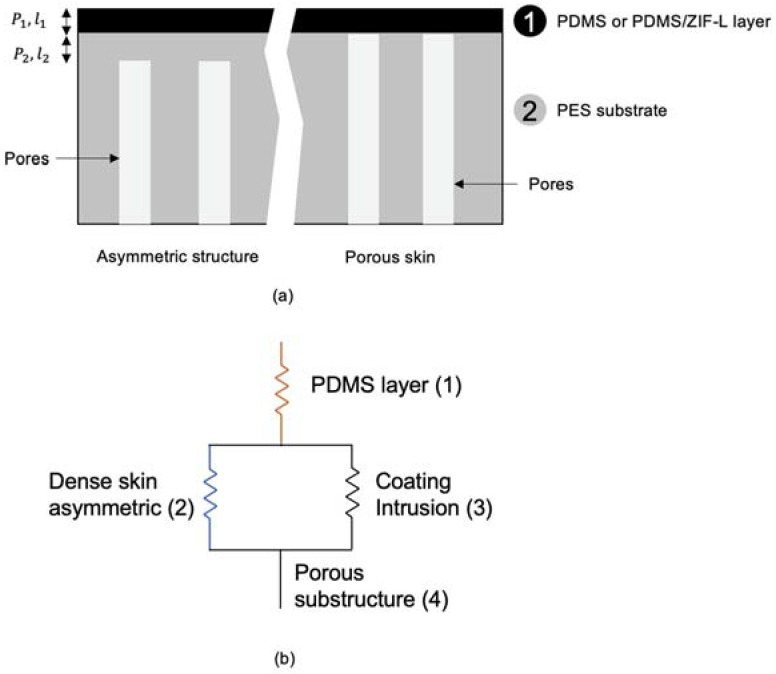
Illustration of (**a**) composite membrane and (**b**) analogous electric circuit.

In 1981, Henis and Tripodi [2] introduced the resistance model to estimate the total permeance of a composite membrane. The model was developed using the analogy of the electricity flow through a series-parallel resistor, as illustrated in Figure 1. The permeance of a gas molecule across the composite membrane is regarded as the inverse of the resistance, which can be calculated by the following equation:

(1) $$R_{i}=\frac{l}{P_{i}A}$$

Based on the electric circuit analogy, the total resistance of the membrane can be written as:

(2) $$R_{T}=R_{1}+\frac{R_{2}R_{3}}{R_{2}+R_{3}}+R_{4}$$

In this study, subscripts 1, 2, 3 and 4 refer to the layer of PDMS or ZIF-L@PDMS layer, thin dense skin of PES, coating layer intrusion in the pores of PES and porous substructure, respectively. The value of *R*_4_ could be neglected due to the minimal resistance for gas permeance possessed by the highly porous substructure. After some simplifications, the permeance ${\left(\frac{P}{l}\right)}_{i}$ of the system was calculated according to the following expression:

(3) Pli=l1P1,i+l2P2,i+P1,iA3A2−1

where A_2_, A_3_, $P_{1,i}$ and $P_{2,i}$ are the area of the dense and porous region of PES membrane, intrinsic permeability of PDMS and intrinsic permeability of PES, respectively. On the other hand, Wang and Chung [3] and Peng et al. [4] took the porosity (ε) of the substrate into consideration, which was somehow similar to the ratio of the dense and porous region of the substrate. Nevertheless, the expression of permeance as a function of ε is:

(4) $${\left(\frac{P}{l}\right)}_{i}={\left(\frac{l_{1}}{P_{1,i}}+\frac{l_{2}}{P_{2,i}+P_{1,i}\left(\varepsilon \right)}\right)}^{-1}$$

In this study, permeance ${\left(\frac{P}{l}\right)}_{i}$ was measured in GPU (10^−6^ cm^3^(STP).cm^−2^ s^−1^ cmHg^−1^) and the permeability *P*_i_ is in the unit of Barrer (1 Barrer = 10^−10^ cm^3^(STP).cm.cm^−2^ s^−1^ cmHg^−1^). Recently, a restriction factor (Ψ) was used by several researchers, including Ramon et al. [5], Wijmans and Hao [6] and Ghadimi et al. [7], to measure the deviation of gas permeance from ideality. Wijmans and Hao [6] calculated the Ψ value using the following equation, as determined by simulation using computational fluid dynamics (CFD):

(5) $$\Psi =\frac{\varepsilon +1.6N_{R}^{1.1}}{1+1.6N_{R}^{1.1}}$$

Meanwhile, $N_{R}$ can be calculated using the following equation:

(6) $$N_{R}=\frac{\tau \varepsilon }{1-\varepsilon }$$

Included in the equation is the dimensionless normalized thickness (*τ*) value, which can be obtained by dividing the thickness of the coating layer by the average pore radius of the substrate. Recently, Hao et al. [8] modified the resistance in series model to accurately predict the permeance of a composite membrane. The resistance model was altered, and the resulting equation is outlined below:

(7) Pli=P1,il11Ψ+l3l1ε−1

where l3l1 is the ratio of pore penetration depth to the coating layer thickness. Based on this equation, the restriction factor is not only equal to the value of Ψ, but the penetration of the coating into the pores of the substrate must be considered. The models presented in Equations (4) and (7) were compared to identify the model best able to describe the performance of ZIF-L@PDMS/PES composite membranes.

## 2. Materials and Methods

### 2.1. Chemicals

Polyethersulfone Ultrason E6020P (PES) and two components Dow Corning Sylgard 184 (PDMS) were used to fabricate the membranes in this work. n-pentane, n-heptane and 1-methyl-2-pyrrolidone which are the solvents used to prepare the membrane were supplied by Merck. Meanwhile, methylimidazole (Sigma Aldrich), zinc nitrate hexahydrate (Sigma Aldrich), triethylamine (Merck) and deionized water were used to synthesize ZIF-L, according to the procedure by Khan et al. [9].

### 2.2. PDMS and ZIF-L@PDMS Flat Sheet Membrane Fabrication

The PDMS and ZIF-L@PDMS flat sheet membranes were fabricated by the solvent evaporation method. Initially, the first component of Sylgard 184 (elastomer) was dissolved in n-pentane at mass ratio of 1:10 and stirred for an hour. In every experiment, 2 g of elastomer was used. The second component of Sylgard 184 (curing agent) was added at the ratio of 1:10 (curing agent:elastomer). The mixture was allowed to mix at 250 rpm for one hour before being poured onto a petri dish (8 cm diameter) that was pre-heated in a water bath at 35 °C. As the n-pentane completely evaporated, the petri dish was transferred to an oven for heating at 100 °C for 12 h. At the end of the process, the petri dish was cooled down and the PDMS membrane was peeled.

**Figure 2:**
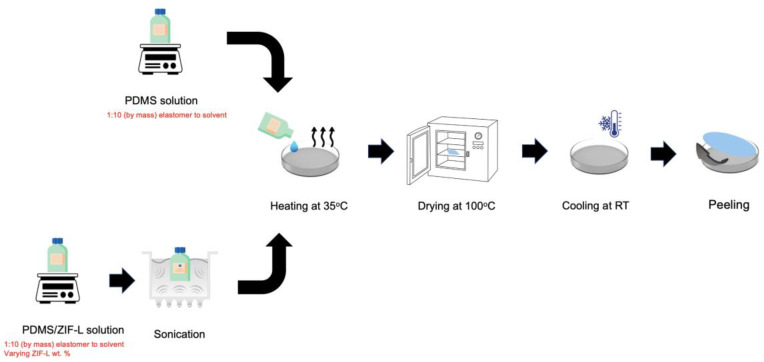
PDMS and ZIF-L@PDMS flat sheet membrane fabrication.

To prepare ZIF-L@PDMS flat sheet membranes, the ZIF-L was introduced in the PDMS solution after the curing agent. Before pouring it onto the petri dish, the solution was sonicated for 45 min at room temperature. The heating procedure followed the pristine PDMS membrane. Figure 2 illustrates the steps to fabricate PDMS and ZIF-L@PDMS membranes.

### 2.3. PES Hollow Fiber Membrane Fabrication

The hollow fiber membrane used in this study was fabricated by the dry jet wet phase inversion method. The dope was first prepared by dissolving PES flakes in NMP. Mixing took place at 60 °C for 12 h. The hollow fiber spinning system was equipped with a pressurized vessel, dope extrusion pump, spinneret, coagulation bath and take-up drum. During the spinning process, the dope was extruded at a ratio of dope extrusion rate to bore fluid flowrate of 3:1; the temperature during spinning and the relative humidity were around 23 °C and 60%, respectively. The nascent fiber traveled at an air gap of 15 cm before approaching the coagulation bath (filtered water at 15 °C). The collected hollow fiber from the take-up drum was then immersed in distilled water for 3 days; water was refreshed daily. Before use, the fibers were air-dried for 3 days.

### 2.4. PDMS/PES and ZIF-L@PDMS/PES Composite Membrane Fabrication

To fabricate PDMS/PES and ZIF-L@PDMS/PES composite membranes, the PDMS solution was first prepared by dissolving the elastomer in n-heptane at a mass ratio of 1:10. After stirring for one hour, the curing agent was added at a ratio of 1:10 (curing agent:elastomer). The stirring continued for another hour before it could be used in the coating process. To prepare the coating solution for ZIF-L@PDMS/PES, the ZIF-L was introduced into the PDMS solution at mass ratio of 1:1 (elastomer:ZIF-L) and the solution was sonicated. The coating time was 120 s and the withdrawal speed was 5 mm/s, unless otherwise specified.

### 2.5. Single Gas Permeation

The gas permeation test was conducted using N_2_, CO_2_ and CH_4_ at a purity of at least 99%. The gases were fed individually, in the order of N_2_, CO_2_ and CH_4_, at 5 bar. There were two modules used in this experiment. To test the performance of a flat sheet membrane, a membrane of 3.14 cm^2^ was placed in a permeation cell. An O-ring and O-shaped rubber sheet were used to secure the membrane in the permeation cell and to prevent gas leakage. Meanwhile, a permeation test for a hollow fiber membrane was carried out using a different module. In each test, a 10 cm membrane was potted to the module using epoxy. The permeance of each gas was calculated using the following equation:

(8) $$\frac{p_{i}}{l}=\frac{Q_{STP}}{A\Delta p}$$

where $\frac{p_{i}}{l}$ is the permeance of the gas in the unit of GPU (10^−6^ cm^3^(STP) cm^−2^ s^−1^ cmHg^−1^), $Q_{STP}$ is the volumetric flow rate of permeate gas at standard temperature and pressure (cm^3^(STP) s^−1^), Δ*p* is the pressure difference across the membrane (cmHg) and *A* is the effective membrane area (cm^2^). Furthermore, the permeability (Barrer) value was calculated by considering the thickness of the membrane, where 1 Barrer is equivalent to 10^−10^ cm^3^(STP) cm cm^−2^ s^−1^ cmHg^−1^. The volumetric flowrate of the permeate was measured using a soap bubble flowmeter. The ideal selectivity values (α) of CO_2_/N_2_ and CO_2_/CH_4_ were calculated according to the following equation:

(9) $$\alpha_{{\mathrm{CO}}_{2}/j}=\frac{\frac{p_{{\mathrm{CO}}_{2}}}{l}}{\frac{p_{j}}{l}}$$

where the subscript *j* refers to N_2_ or CH_4_ to calculate the selectivity of CO_2_/N_2_ and CO_2_/CH_4_, respectively.

### 2.6. Sample Characterization

The surface cross-sectional images of the membranes were obtained using scanning electron microscopes (HITACHI Tabletop Microscope instrument (TM-3000, Tokyo, Japan) operated at 15 kV. Each sample was freeze-fractured using liquid nitrogen for a clean-cut of the sample for cross-sectional analysis. To analyze the surface porosity of a membrane, ImageJ software was used. On the other hand, the PDMS layer thickness was obtained using the gravimetric method [10]. Additionally, thermogravimetric (TGA) analysis was carried out to identify the weight of ZIF-L adhered to the composite membrane. The sample was subjected to heating under nitrogen, up to 800 °C where the heating rate was fixed to 10 °C/min.

## 3. Results and Discussion

### 3.1. ZIF-L@PDMS Membrane Performance

**Figure 3:**
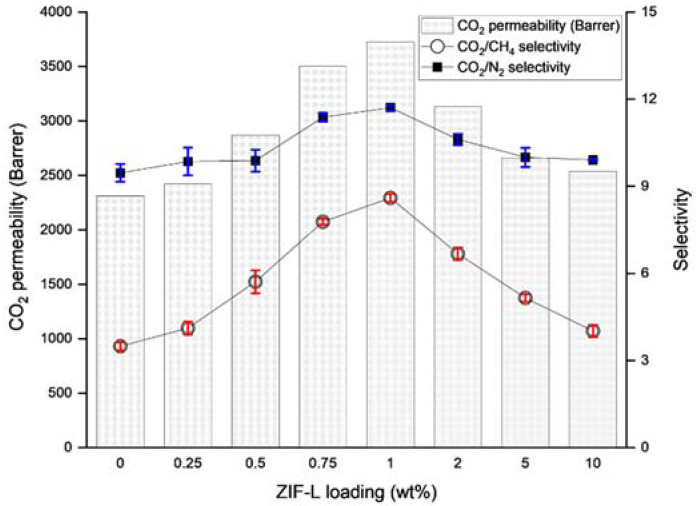
Separation performance of ZIF-L@PDMS membrane at various loadings of ZIF-L.

The performance data for the ZIF-L@PDMS flat sheet membranes are shown in Figure 3. As is clear from the graph, the CO_2_ permeability and selectivity increased steadily up until 1 wt%. Without ZIF-L, the pristine PDMS membrane recorded 2310 Barrer of CO_2_ permeability, while the CO_2_/N_2_ and CO_2_/CH_4_ selectivity values were 9.46 and 3.49, respectively. At 1 wt% of ZIF-L, the permeability of CO_2_ increased by 61.3%, while the selectivity of CO_2_/N_2_ and CO_2_/CH_4_ increased by 23.8% and 105.4%, respectively. These observations were consistent with other types of polymers, including polyimide [11] and PES [12]. Evidently, the performance enhancement can be attributed to the characteristics of ZIF-L, which is highly selective toward CO_2_. With its pore size close to the kinetic diameter of CO_2_, only CO_2_ was able to pass through ZIF-L, while N_2_ and CH_4_ were forced to travel a tortuous path across the membrane to permeate. Moreover, Chen et al. [13] claimed that the cushion-shaped cavity of ZIF-L was unique and well-suited to accommodate CO_2_ molecules.

**Figure 4:**
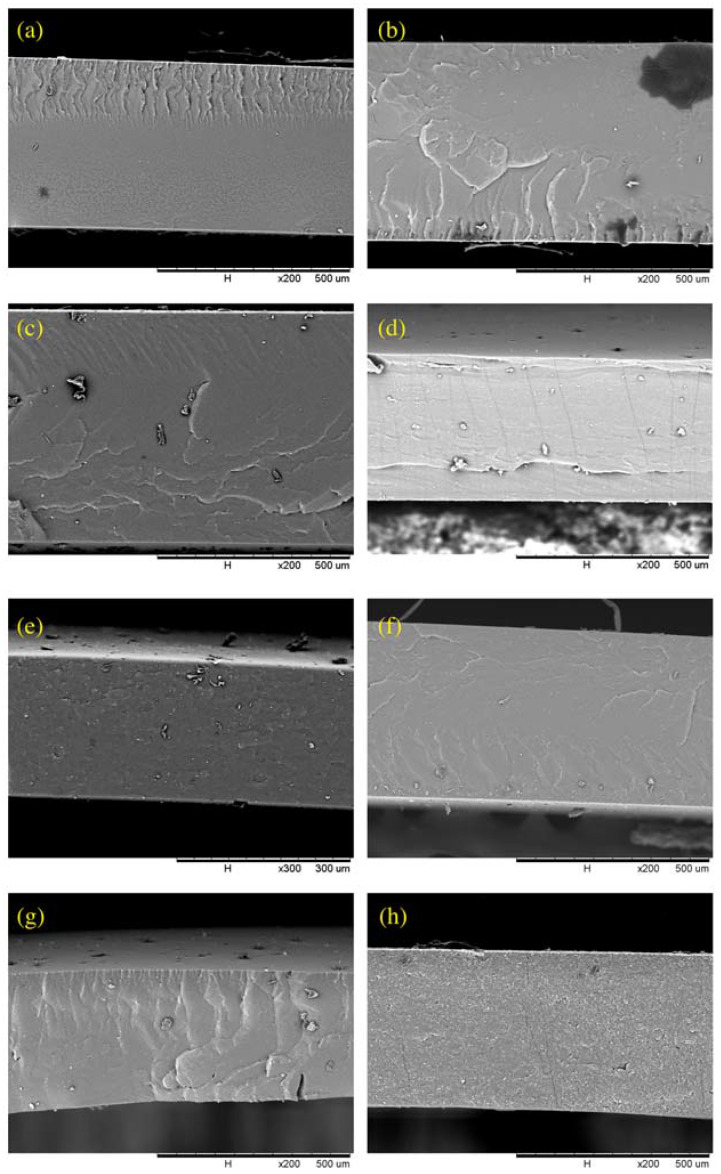
Cross-sectional images of ZIF-L@PDMS flat sheet membrane at ZIF-L loading of (**a**) 0 (**b**) 0.25 (**c**) 0.50 (**d**) 0.75 (**e**) 1.0 (**f**) 2.0 (**g**) 5.0 and (**h**) 10.0 wt%.

The cross-sectional images of the PDMS and ZIF-L@PDMS flat sheet membranes are shown in Figure 4. Based on the SEM images, ZIF-L was found to be uniformly distributed in the membrane, even though agglomeration of ZIF-L could be spotted at high loading. Meanwhile, the decline of CO_2_ permeability was recorded at high ZIF-L loading due to an enhanced resistance for gas permeation created by the high amount of ZIF-L. The drop in selectivity that accompanied this situation could also have been caused by interfacial defects due to the poor interaction between the polymer and ZIF-L. Nevertheless, the selectivity did not drop below the intrinsic value of the PDMS.

**Figure 5:**
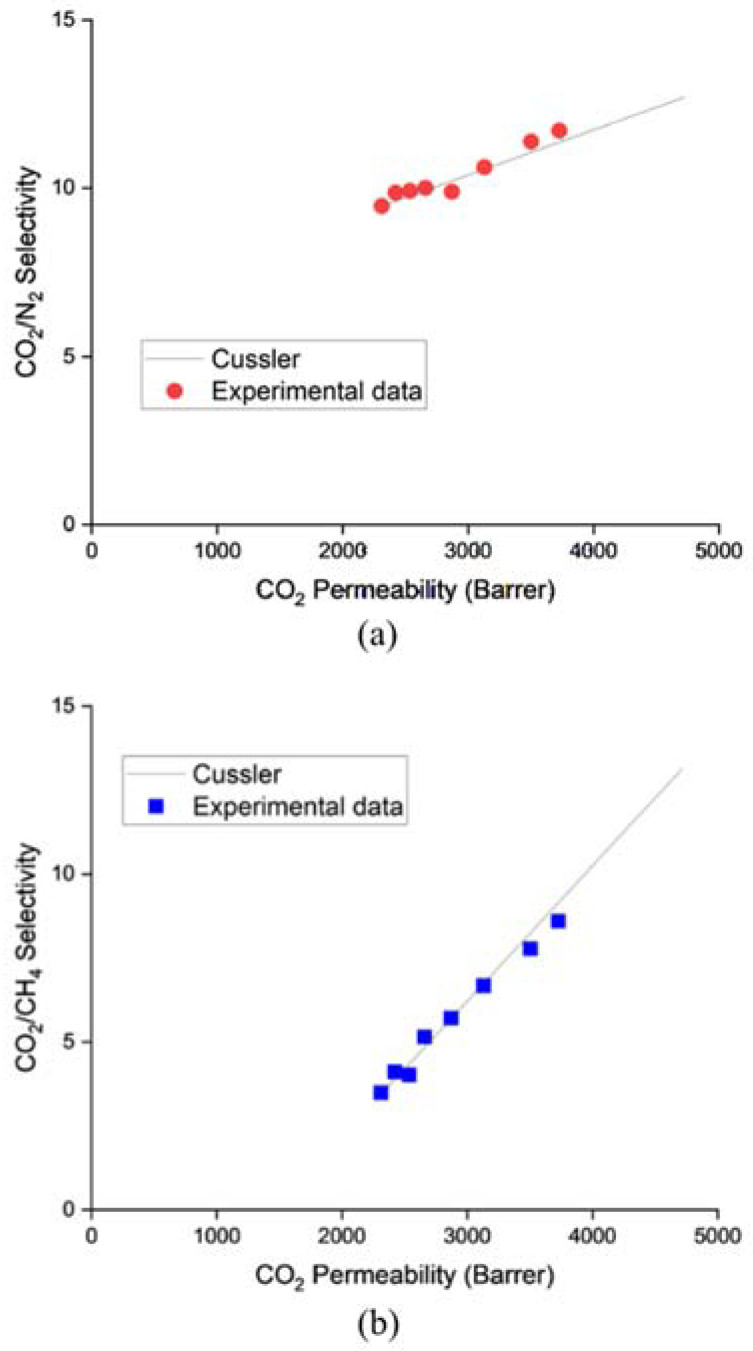
Plot of Cussler model and experimental data obtained in this study for (**a**) CO_2_/N_2_ and (**b**) CO_2_/CH_4_ gas pair.

More importantly, the ZIF-L@PDMS flat sheet membrane was fabricated to confirm that the model fit the experimental data. The ZIF-L@PDMS was regarded as a mixed matrix membrane (MMM), located on the skin of membrane support. Over the years, multiple models have been introduced to predict the performance of mixed matrix membranes. Widely known models include Maxwell, Bruggeman and Cussler. However, the first two were not considered for an MMM developed using nanosheet as a filler, as they did not take shape and filler size into consideration. Meanwhile, Cussler included the aspect ratio in the model to accurately predict the performance of MMMs that contained nanosheets. A detailed analysis by Sheffel and Tsapatsis [14] revealed that the aspect ratio was a crucial parameter, especially at high values. The equation is as given below:

(10) $$\frac{P_{o}{}_{i}}{P_{i}}=\frac{1}{1-\theta +\frac{1}{\frac{1}{\delta \theta }+\frac{1-\theta }{\alpha^{2}\theta^{2}}}}$$

where $P_{o}{}_{i}$ is the permeability of pristine PDMS, $P_{i}$ is the permeability of ZIF-L@PDMS, θ is the volume fraction of ZIF-L, δ is the ratio of permeability between the ZIF-L and pristine PDMS, while α is the aspect ratio of the ZIF-L. The ZIF-L used in this study had an aspect ratio of 31 and its permeability was obtained by back-calculation method at 10 wt% of ZIF-L loading [15] using the Cussler model. The straight line in Figure 5a,b represents the permeability and selectivity, as predicted by the Cussler model, while the red and blue marks are the experimental data from Figure 3. Based on the plot, the experimental data fitted the Cussler model, hence, that model was deemed suitable to be used to predict the overall performance of ZIF-L@PDMS/PES composite membranes.

### 3.2. PDMS/PES Composite Membrane

**Figure 6:**
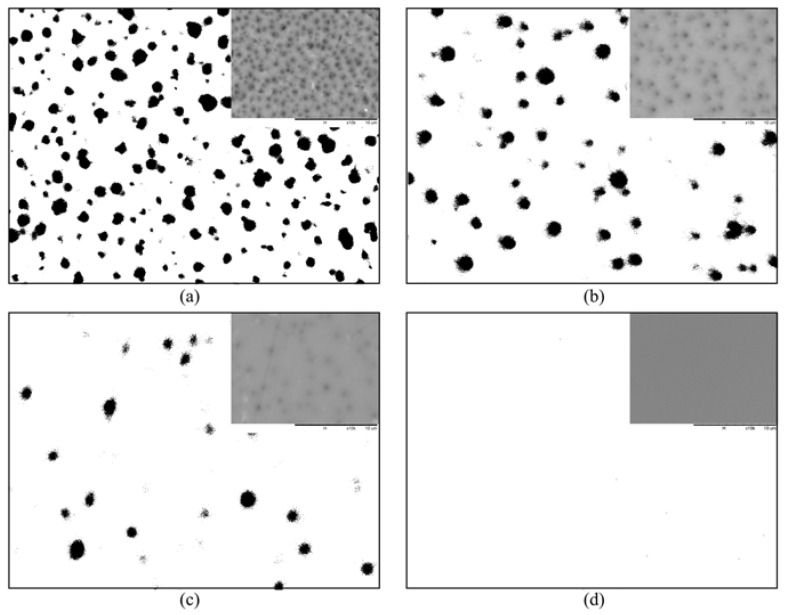
Surface morphology of substrate at (**a**) 21 wt% (**b**) 25 wt% (**c**) 27 wt% and (**d**) 29 wt% PES.

First, the characteristics of the hollow fiber substrate at various PES concentrations were determined. Various porosity was achieved by preparing the membrane at different dope concentrations. The SEM images can be found in Figure 6. As shown, their surface morphologies were analyzed using ImageJ software. It was obvious that the surface porosity of the substrate was high at lower PES concentrations, due to the influence of demixing rates and the strong solvent used during membrane fabrication [16]. Meanwhile, the porosity and mean surface pore size data are provided in Table 1. According to the analysis, the surface porosity of the membrane decreased steadily as a function of PES concentration, while the pore size showed the opposite trend. Nevertheless, at any PES concentration, the pore size was large enough to easily allow gases with large kinetic diameters to pass through the membrane.

**Figure 7:**
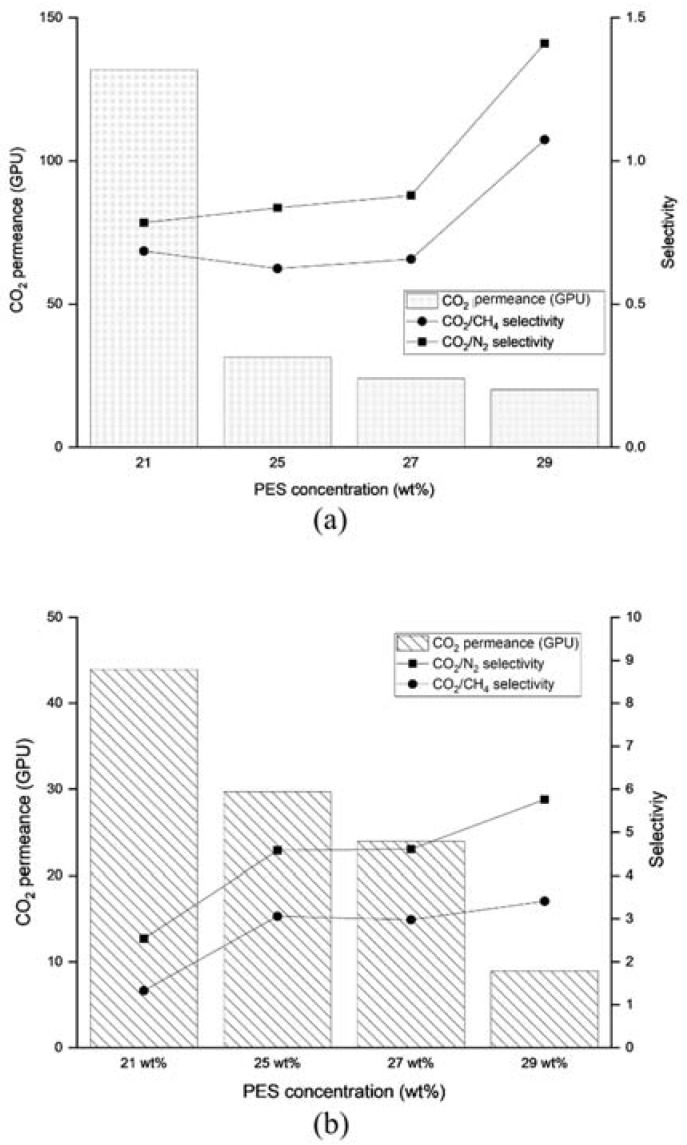
Separation performance of (**a**) uncoated PES substrate and (**b**) coated PES.

Figure 7a provides performance data on the PES substrate at various concentrations. The permeance of the gas reduced significantly at 29 wt% PES, as this concentration was identified as the critical concentration that allowed the formation of thicker dense layers and minimal porosity [17]. Without the dense skin, the gas permeated at high flux with no selectivity. The permeance value of the uncoated PES substrate was much higher, compared to the coated PES substrate with PDMS, as seen in Figure 7b. That observation justified the assumption made to neglect the R_4_ term from the resistance model. According to Henis and Tripodi [2], the porous substructure indeed caused resistance for the gas permeation; however, the resistance of the matrix (which was similar for fast and slow gases) represented a much larger fraction of the total resistance for the fast gas than for the slow gas. Although a dense layer was formed at 29 wt% PES, the membrane exhibited low selectivity, typically due to pinhole formation and defects. An asymmetric membrane is defined as defect-free if the ideal selectivity of the membrane is higher than 80% of the intrinsic value [18]. Apparently, this was not applicable to the PES hollow fiber membrane in this work, based on the permeance data provided in Figure 7b. According to Zulhairun et al. [19], the formation of defects are almost inevitable. However, for many years, the dip coating method has been used to seal pinholes and improve the performance of hollow fiber membranes.

**Table 1:** Characteristics of PES substrate and PDMS layer thickness of PDMS/PES membrane.

PES (wt.%)	Surface Porosity (ε)	Mean Surface Pore Size (nm)	PDMS Layer Thickness (cm)
21	0.230	231.05	0.00065
25	0.052	233.38	0.00061
27	0.020	253.05	0.00059
29	0.000092 ^1^	-	0.00033

^1^ Data calculated by backward calculation according to Wang and Chung [3] using N_2_ and CH_4_ permeance data. Permeance of pristine PES data obtained from Chen et al. [20]. The dense layer of PES substrate at 29 wt% PES is 5.87 × 10^−6^ cm.

The influence of PES concentration—and hence, the membrane’s surface porosity—on CO_2_ permeance can be viewed in Figure 7a,b. As shown, permeance was obviously dependent on the porosity of the PES substrate. The trend of the graph suggested that permeance declined steadily with porosity. This was attributed to the funnel effect’s greater intensity due to the lower number of pores, as revealed by Ramon et al. [5] in a composite membrane. Additionally, the thickness of the PDMS layer was affected by the porosity of the substrate, as demonstrated in Table 1. The thickness of the coating layer indeed influenced the funnel effect on the interface of the substrate and coating.

### 3.3. Prediction via Resistance in Series Model

**Figure 8:**
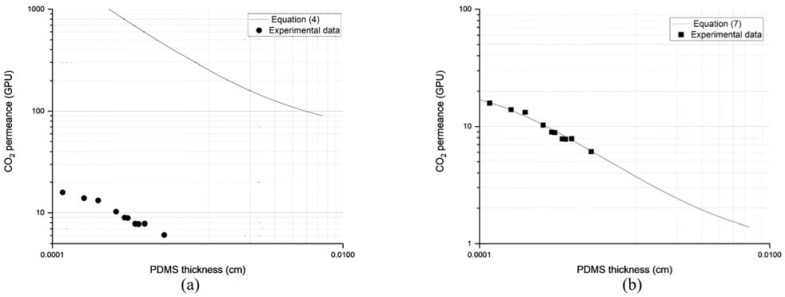
Comparison of experimental data of CO_2_ permeance of PDMS/PES composite membrane with (**a**) Equation (4) and (**b**) Equation (7).

Figure 8 shows a comparison of the experimental data of the CO_2_ permeance of PDMS/PES composite membrane with two models that considered porosity in the equation. Experiments were carried out using 29 wt% PES hollow fiber as the substrate. The characteristics of the membrane can be found in Table 1. In this study, various PDMS layer thicknesses were formed by manipulating the withdrawal speed during the coating process.

As seen in Figure 8a, Equation (4) was unable to provide a good estimation of the CO_2_ permeance by only considering porosity. The experimental data deviated significantly from the model. The overestimation could have been due to the penetration of the top layer material into the pores, as simulated by Hao et al. [8]. As explained by several other research groups, the gas transport took place primarily at the pore of the substrate. This is known as the funnel effect. Low porosity of a substrate also resulted in a less active area for the gas to be transported to the other side [5].

On the other hand, the prediction by Equation (7) was made by first calculating the ratio of l3l1. It was done by back calculation, using data on ideal and actual CO_2_ permeance, as well as surface porosity, as provided in Table 1 at Ψ, and τ values of 0.045 and 436.96, respectively. The ratio of l3l1 was considered constant (0.0052) at any withdrawal speed during the coating. The availability of such data allowed Equation (7) to be plotted, as shown in Figure 8b. Interestingly, the inclusion of pore penetration into the model facilitated prediction of CO_2_ permeance with good accuracy. This observation was also supported by Hao et al. [8] for track-etched substrates. Moving forward, Equation (10) was inserted in Equation (7) to allow the CO_2_ permeance prediction across ZIF-L@PDMS/PES composite membranes at specific loadings of ZIF-L. The term $\frac{P_{1,i}}{l_{1}}$ on the right-hand side of Equation (7) was first calculated using Equation (10). The extension of the equation was indeed necessary, as the coating layer contained inorganic fillers that could be considered a mixed matrix membrane. In this regard, the overall composite membrane performance depended on the loading of ZIF-L on the coating layer, as data in Figure 3 suggest.

### 3.4. ZIF-L@PDMS/PES Composite Membrane

**Figure 9:**
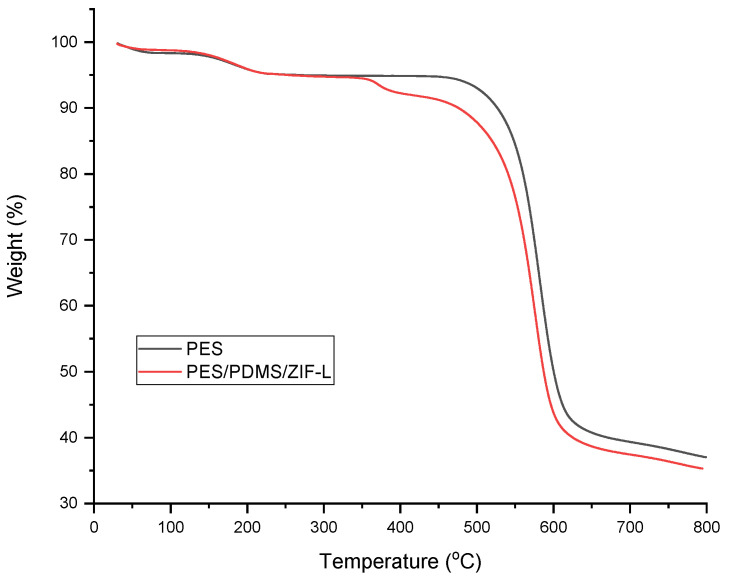
TGA analysis of PES and ZIF-L@PDMS/PES membranes.

To predict the CO_2_ permeance of ZIF-L@PDMS/PES composite membrane, the weight of ZIF-L, adhered to the membrane, was determined. TGA analysis, shown in Figure 9, was helpful in estimating the fraction of ZIF-L in the composite membrane. Based on mass balance, the fraction of ZIF-L in the composite was estimated using the method outlined by Francavilla et al. [21], wherein the sample residue was considered in the calculation. The thermal analysis suggested a ratio of elastomer: ZIF-L of 1:1, the fraction of ZIF-L in the composite membrane was 1.82 wt%.

The combination of Equations (7) and (10) predicted that the CO_2_ permeance at such ZIF-L loading would be 9.62 GPU. The prediction was made based on the assumption that the thickness of the ZIF-L@PDMS layer formed on the substrate was identical to the pristine PDMS, as provided in Table 1, and that the ratio of l3l1 was 0.0052. To validate the model, the ZIF-L@PDMS/PES composite membrane was subjected to CO_2_ at 5 bar. The performance data are tabulated in Table 2. The experimental CO_2_ permeance data recorded a very close value to the prediction, with minimal error.

For comparison purposes, Equation (4) was also used to estimate the permeance of CO_2_ across the ZIF-L@PDMS/PES composite membrane. The equation predicted that the CO_2_ permeance would be 801.21 GPU, which resulted in a large error compared to the experimental data.

## 4. Conclusions

The best model to predict the permeance of CO_2_ across the composite ZIF-L@PDMS/PES was identified in this work. To accurately predict the permeance of CO_2_ across such composite membranes, the penetration of the coating layer must be taken into consideration. Moreover, the presence of ZIF-L in the composite membrane requires the consideration of inorganic loading in the model. Equation (7) was further extended by including the Cussler model, which permitted greater accuracy in predicting the CO_2_ permeance across a ZIF-L@PDMS layer. Finally, the model had only minimal error between the predicted and experimental data, at 0.7%. Hollow fiber membranes are regarded as an appropriate option for industrial use. Thus, a suitable model, able to predict their performance, must be determined. In this work, the model’s predictions fit with the experimental CO_2_ permeance data collected. Accordingly, after a scaling-up process, we postulated that it could work as well at the industrial scale.

## Figures and Tables

**Table 2:** Predicted and experimental data of CO_2_ permeance in ZIF-L@PDMS/PES composite membrane.

	Permeance (GPU)	Selectivity		Error		
		CO_2_/N_2_	CO_2_/CH_4_	Permeance	Selectivity	
					CO_2_/N_2_	CO_2_/CH_4_
Predicted values	9.62 GPU ^1^ 801.21 GPU ^2^	9.98	3.82	-	-	-
Experimental data	9.69 GPU	10.96	4.22	0.7% 98.8%	8.9%	9.4%

^1^ calculated using Equations (7) and (10). ^2^ calculated using Equation (4).

## Data Availability

Not applicable.
